# Precipitation and potential evapotranspiration determine the distribution patterns of threatened plant species in Sichuan Province, China

**DOI:** 10.1038/s41598-022-26171-5

**Published:** 2022-12-27

**Authors:** Jiangong Li, Bikram Pandey, Mohammed A. Dakhil, Manita Khanal, Kaiwen Pan

**Affiliations:** 1 Key Laboratory of Forest Ecology in Tibet Plateau, Ministry of Education, Institute of Tibet Plateau Ecology Research, Tibet Agricultural and Animal Husbandry University, Nyingchi, 860000 Tibet People’s Republic of China; 2 National Forest Ecosystem Observation and Research Station of Nyingchi Tibet, Nyingchi, 860000 Tibet People’s Republic of China; 3 CAS Key Laboratory of Mountain Ecological Restoration and Bio-Resource Utilization and Ecological Restoration Biodiversity Conservation Key Laboratory of Sichuan Province, Chengdu Institute of Biology, Chinese Academy of Sciences, Chengdu, 610041 Sichuan People’s Republic of China; 4 University of Chinese Academy of Sciences, Beijing, People’s Republic of China; 5 Botany and Microbiology Department, Faculty of Science, Helwan University, Cairo, 11795 Egypt; 6 Centre for Applied Ecology, Research Network in Biodiversity and Evolutionary Biology (CEABN, InBIO), School of Agriculture, University of Lisbon (ISA, UL), Tapada da Ajuda, 1349-017 Lisboa, Portugal; 7 Institute of Forestry, Tribhuvan University, Pokhara, 33700 Nepal

**Keywords:** Biodiversity, Biogeography, Ecological modelling, Theoretical ecology

## Abstract

A fundamental goal of ecologists is to determine the large-scale gradients in species richness. The threatened plants are the priority of such studies because of their narrow distribution and confinement to a specific habitat. Studying the distribution patterns of threatened plants is crucial for identifying global conservation prioritization. In this study, the richness pattern of threatened plant species along spatial and elevation gradients in Sichuan Province of China was investigated, considering climatic, habitat-heterogeneity (HHET), geometric constraint and human-induced factors. The species richness pattern was analyzed, and the predictor variables, including mean annual temperature (MAT), mean annual precipitation (MAP), potential evapotranspiration (PET), HHET, and disturbance (DIST), to species richness were linked using the geographical distribution data of threatened species compiled at a spatial resolution of 20 km × 20 km. Generalized linear models and structural equation modelling were used to determine the individual and combined effects of each variable on species richness patterns. Results showed a total of 137 threatened plant species were distributed between 200 and 4800 m.a.s.l. The central region of the province harbors the highest species diversity. MAP and PET profoundly explained the richness pattern. Moreover, the significant role of DIST in the richness patterns of threatened plants was elucidated. These findings could help determine the richness pattern of threatened plant species in other mountainous regions of the world, with consideration of the impact of climate change.

## Introduction

The study of distribution patterns of threatened species has great significance to biodiversity conservation^1^. Understanding the distribution of plant species has strong implications for conservation^2–4^. Therefore, species richness pattern and the mechanisms that determine species distribution have long fascinated ecologists and bio-geographers^3,5,6^. Richness patterns could be studied at spatial and elevation gradients. The most common pattern of plant species richness along the mountain gradients manifests into four forms: increasing, decreasing, U-shaped (few species at mid-elevation), and unimodal (higher number of species at mid-elevation) patterns^7^. A study in Nepal showed that threatened plant species follow a linear decline in their richness pattern along elevation^1^. Meanwhile, uncertainty remains regarding the spatial distribution pattern of threatened plant species, especially in diversity-rich regions of China. For example, a recent study revealed that with the increase in human activities and climate change, plants in higher elevations are at risk of extinction^8,9^. Therefore, effective and sustainable protection of biodiversity has become an important concern of ecologists and conservationists.

Multiple variables, namely, climatic, anthropogenic disturbances (DISTs), and mountain topography, may affect the distribution of species in a large geographical area. Climate change, rapid increase in population, destruction of vegetation, and fragmentation of natural habitats all contribute to biodiversity decline^10^. Bhattarai and Vetaas^11^ identified a complex phenomenon of climatic variables relative to elevation changes in a mountainous region of the Himalayas. Therefore, to understand the driving mechanisms that determine the richness pattern of plants, studies should focus on variables that have a direct influence on the growth of plants^12,13^. Climatic variables, such as temperature, precipitation, and potential evapotranspiration (PET) are the most powerful predictors that determine the richness pattern of plants^5,11,13–15^. Temperature and water have a direct relationship with the pattern of species richness by accelerating the metabolic activities in plants^12,16^. Similarly, PET has been closely linked to water–energy dynamics, considered the most influential variable driving the distribution pattern in plants^11^. Meanwhile, variation in species diversity of threatened plants is mostly related to anthropogenic activities, such as land-use change, urbanization, road construction, industrialization, and fragmentation of habitat. Moreover, habitat heterogeneity (HHET) increases habitat diversity, thus increasing species occurrence and governing species richness gradients by local and regional species turnover^17–19^. The variability in habitat creates high niche diversity that allows species to coexist^5,20^. The mid-domain effect (MDE) hypothesis is another vital mechanism related to understanding the effect of mountain constraints on richness patterns^21,22^. According to the MDE hypothesis, the mid-elevation peak in species richness is the result of the “geometric constraints” of a mountain. These “geometric constraints” may lead to the overlapping of species near the midpoint of a mountain, causing the richness to peak at mid-elevation. Further, the MDE states that the diversity in species richness in mountain domains is independent of any environmental gradients and that it represents a “null model” for diversity patterns^21,22^. Some studies have validated the effect of the MDE in the mountains of China^23–25^. In the present paper, the effects of temperature, precipitation, PET, DIST, and the MDE responsible for the richness pattern of threatened plants in Sichuan Province were examined.

**Figure 1:**
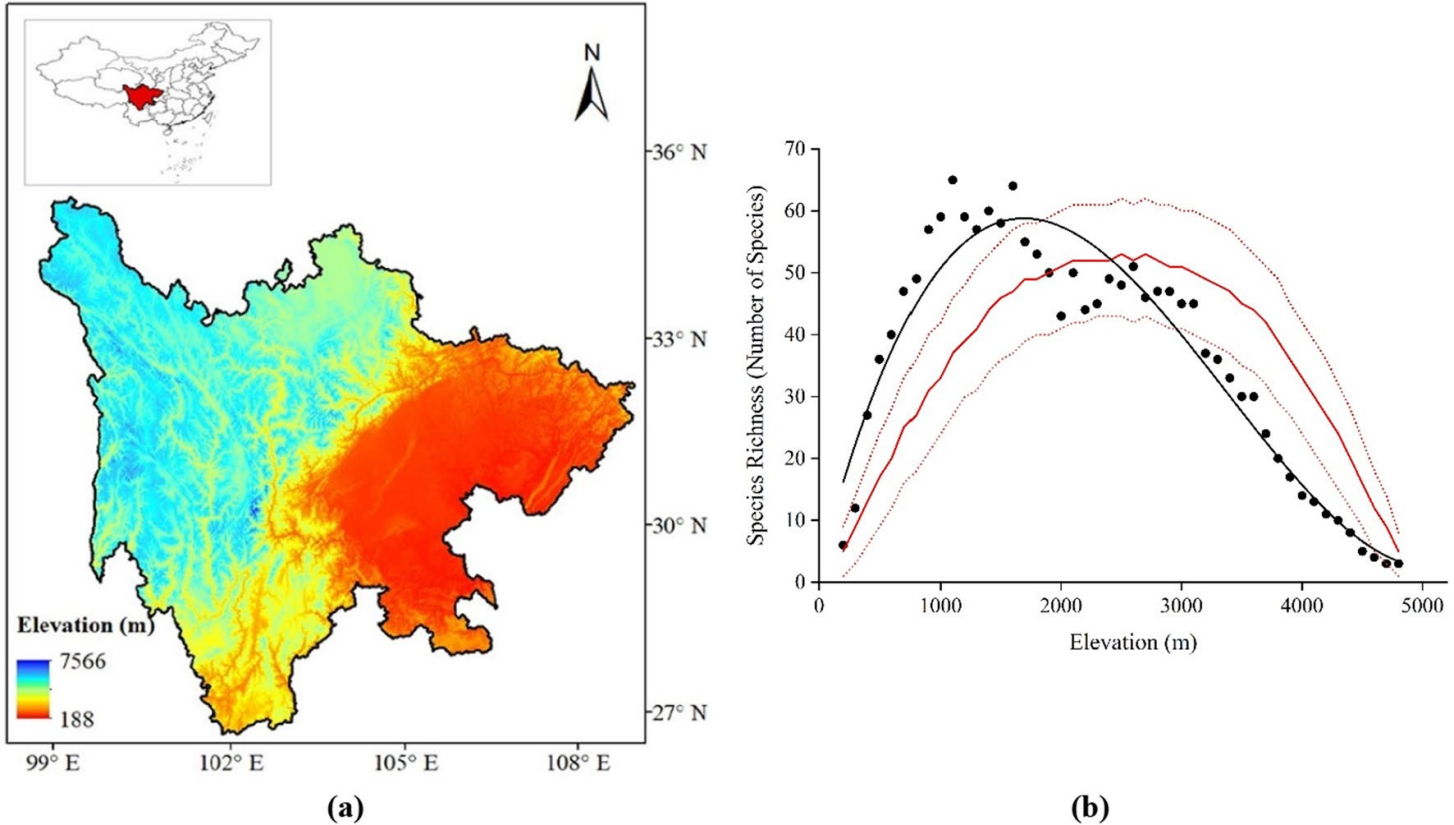
(**a**) Map of Sichuan Province (study area) showing elevation gradient. (**b**) Relationship between species richness of threatened plants and the MDE in Sichuan Province of China. Black solid dot represents the species richness while the black line is the highest fit of the polynomial curve. The explanatory power of the regression model is significant at *p* < 0.005. The red solid line is the predicted mean richness derived from RangeModel. Red dotted line show 95% confidence intervals predicted by the mid-domain effect (MDE) randomization model. (**a**) was generated in ArcGIS (version 10.3.1) using Shuttle Radar Topography Mission (SRTM)^28^ digital elevation data. The map of China and Sichuan were generated from the standard map of drawing number GS (2019) 1698, downloaded from the official website of the National Administration of Surveying, Mapping and Geoinformation of China (NASG).

Sichuan Province is located in southwestern China (Fig. 1a) within the transition zone between the Qinghai–Tibetan Plateau and the plain of the middle and lower reaches of the Yangtze River. The topography and climate in Sichuan Province are complex and diverse, forming variations and complexities in vegetation types^26^. The geomorphology of this province varies greatly from east to west. The average elevation also increases from east to west. The variation in climatic and biological factors in this region have favored high floristic richness. Around 9254 species of vascular plants belonging to 1621 genera and 232 families could be found in Sichuan Province^26^. Almost one-third of estimated vascular plant species reported from China are found in this region. Variation in plant diversity in response to environment plays an important roles in ecosystem services at global and national scales, including the ecological transition zone in the upper reaches of the Yangtze River, one of the global biodiversity hotspots and home to the wild giant panda population^27^. Despite the large number of ecological studies conducted in high mountainous regions, a comprehensive study on the distribution pattern of threatened plants in one of the most important ecoregions of China has not been conducted.

Although the distribution patterns of plant species have been extensively explored over the past several decades^3,5,8,18,29,30^, none of the studies prioritized the evaluation of the richness patterns of threatened plant species along spatial gradients. Plants are threatened not only because of human influence but also by climatic factors^1^. Therefore, in the present study, the relationships among elevations, climatic, “geometric constraints,” and human influence were explored by investigating the variation in threatened plant species diversity across Sichuan Province, China. The objectives were as follows: (i) to explore the elevation-richness relationships among threatened plant species along an elevation gradient in Sichuan Province, (ii) to evaluate the role of the MDE in the richness pattern of threatened species, and (iii) to evaluate the effect of climatic characteristics, HHET and DIST on the species diversity of threatened plants. To our knowledge, this study was the first to investigate the role of climatic, the MDE, HHET, and DIST on threatened plants in an ecologically diverse province of China.

## Results

### Distribution of threatened plant species in Sichuan

A total of 137 threatened species were found to be distributed between 200 and 4800 m above sea level in the Sichuan Province, China. The polynomial regression analysis showed a declining trend along the elevation gradients (*R*^2^ = 0.942, p < 0.005, Fig. 1b). The number of species tended to increase between 200 and 1100 m, forming a hump at 1000 m, then decline sharply up to 4800 m. The highest number of species was recorded at 1100 m (n = 65), and the least number of species was reported at 4800 m (n = 2). The MDE explained the least variation in the richness pattern of threatened plant species along the elevation gradient (*R*^2^ = 0.213, *p* < *0.013*, Fig. 1b).

**Figure 2:**
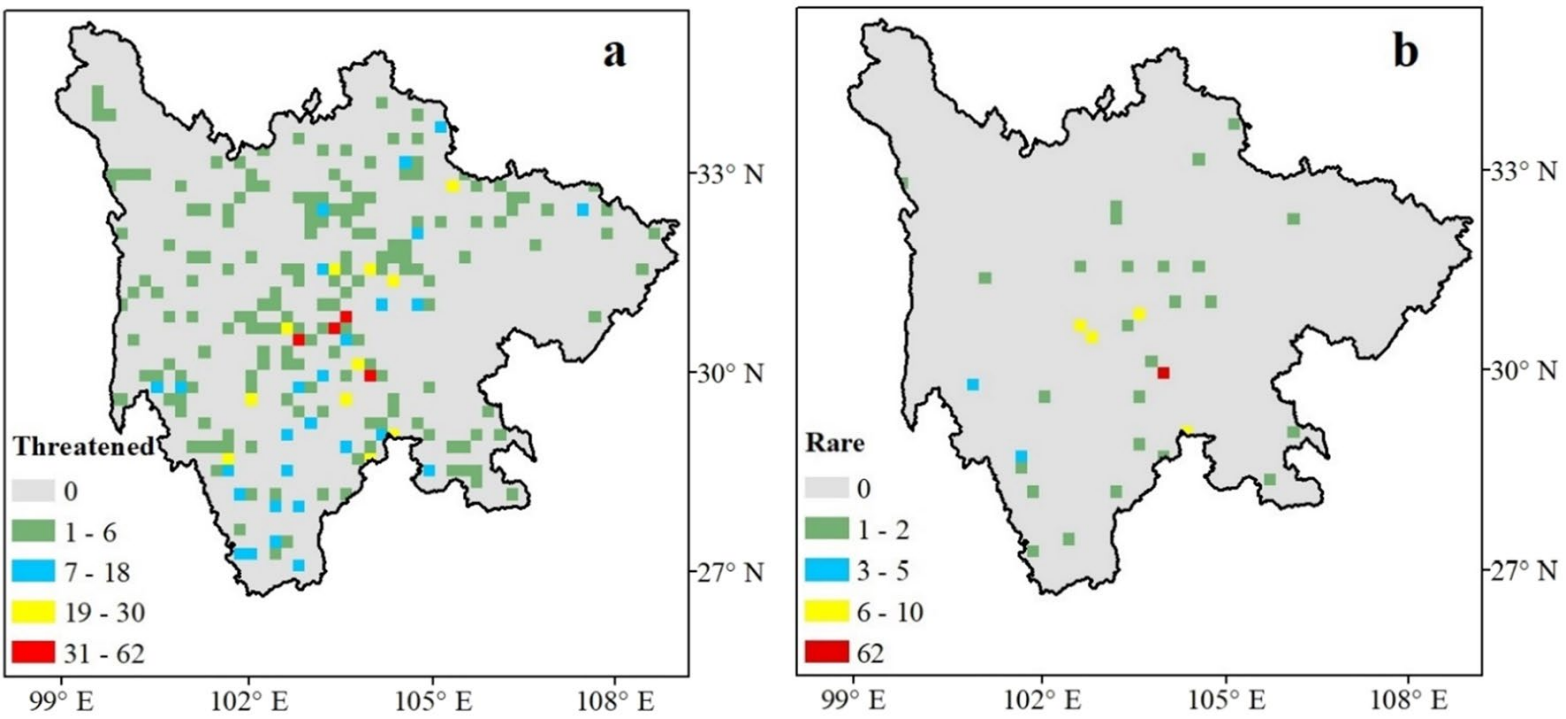
Map of species richness of all study threatened species (**a**) and hotspots of threatened species (**b**). The red grids in (**a**) are the centre of threatened plant richness in Sichuan. All the figures were generated in ArcGIS (Version 10.3.1).

Along the 20 km × 20 km grid cells, 134 threatened species were found in 223 grids. The threatened plant richness ranged from 1 to 62 (mean 4.66 ± 0.49 SE; Fig. 2a). The distribution of threatened plants in Sichuan was uneven. However, the center of threatened plant richness was identified based on the output map, i.e., Central Sichuan. The central region of the province harbored the highest species distribution. Moreover, part of the Hengduan Mountains region (27° N and 102° E) was characterized by a rich diversity of threatened plant species. Similarly, Central Sichuan was found to be a hotspot for threatened plant species (Fig. 2b).

### Factors determining the richness pattern of threatened plant species

**Figure 3:**
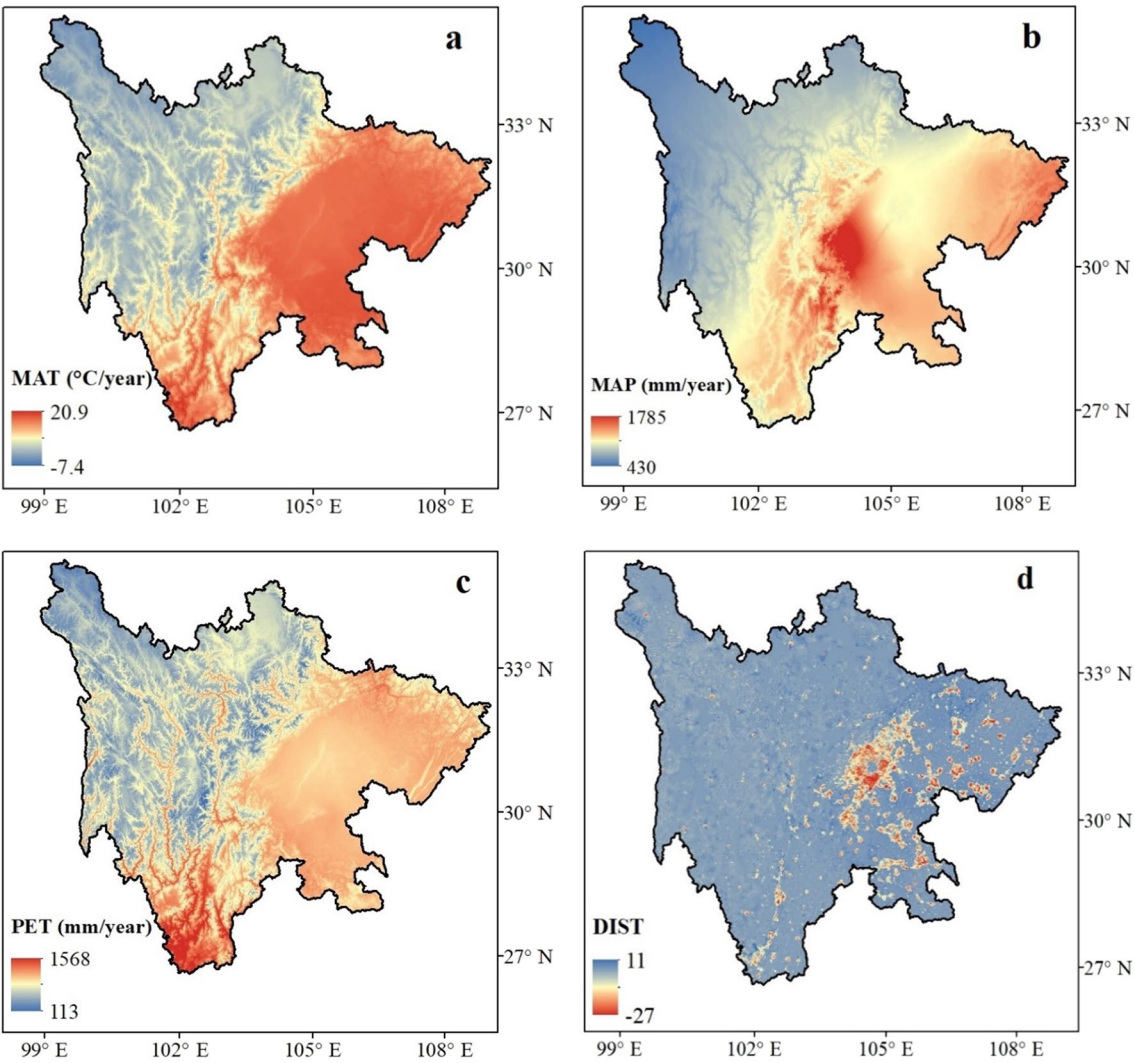
Map showing the differentiation in impact variables in the study area: (**a**) mean annual temperature (MAT), (**b**) mean annual precipitation (MAP), (**c**) potential evapotranspiration (PET), and (**d**) disturbance (DIST). All the figures were generated in ArcGIS (Version 10.3.1). (**a**) and (**b**) were produced using CHELSA climate data^31^, (**c**) using MOD16 data^32^, and (**d**) using Human footprint index^33^.

The study area was found to have a MAT ranging between − 7.4 °C and 20.9 °C (Fig. 3a). The eastern region of the province experiences warmer temperatures than the western region. Meanwhile, the eastern region showed the maximum MAP (1785 mm) compared with the western region (430 mm, Fig. 3b). A similar effect of PET was found in the study area, ranging between 113 and 1568 mm/year (Fig. 3c). The highest rate of DIST was found in the eastern region. The positive values indicated no disturbance, while the negative values show the degree of disturbance (Fig. 3d).

All the variables showed a significant relationship with the richness pattern of the threatened plant species. The *Adj R*^2^ values of the models ranged between 54.71 and 89.22% (Table 1). The variables that best explained the richness pattern of threatened species in Sichuan were PET, MAP, and DIST. The combined model developed using stepwise GLM regression showed significant effects of the three variables (*p* < 0.001, *Adj R*^2^% = 89.22), with the least AIC value (AIC = 1118.2). MAT and PET explained 85.47% deviance in the richness pattern of threatened species in Sichuan. A similar effect was noticed when PET and MAP were used as impact variables explaining 85.04% deviance in the richness pattern of threatened species (Table 1). For the individual predictor of species richness, PET showed a significant positive relationship with species richness explaining 84.37% (*p* < 0.001, AIC = 1126.5) deviance (Table 1), followed by MAT, which explained 66.22% (*p* < 0.001) deviance. A notable detail is that DIST was also one of the major variables that showed a significant relationship with the richness of threatened plant species in Sichuan (*p* < 0.001, *Adj R*^2^% = 58.61, AIC = 1177.8) (Table 1).

**Table 1:** Summary of the regression statistics for variable combinations including MAT, PET, MAP, HHET and DIST on species richness of threatened plant species.

Variables included in the best model	*P-values*	*Adj R*^2^ (%)	AIC
PET (+ 0.38), MAP (+ 0.14), DIST (− 0.24)	< 0.001	89.22	1118.2
MAT (− 0.12), PET (+ 0.84)	< 0.001	85.47	1123.0
PET (+ 0.73), MAP (− 0.006)	< 0.001	85.04	1124.8
PET (+ 0.73)	< 0.05	84.37	1126.5
PET (+ 0.05), DIST (+ 0.001)	< 0.05	78.81	1138.3
MAP (+ 0.21), DIST (− 0.02)	< 0.05	72.00	1147.4
MAT (+ 0.67)	< 0.001	66.22	1153.2
HHET (+ 0.18)	< 0.05	60.13	1170.9
DIST (− 0.28)	< 0.001	58.61	1177.8
MAP (+ 0.54)	< 0.001	54.71	1187.2

The best model were selected based on high *adj R*^2^ and low AIC values. *P-value* denotes the significant of a model. Numbers in parentheses are coefficients of respective variables. All the variables in the model have VIF < 5.

MAT: mean annual temperature; PET: potential evapotranspiration; MAP: mean annual precipitation; HHET: Habitat-heterogeneity; DIST: disturbance.

**Figure 4:**
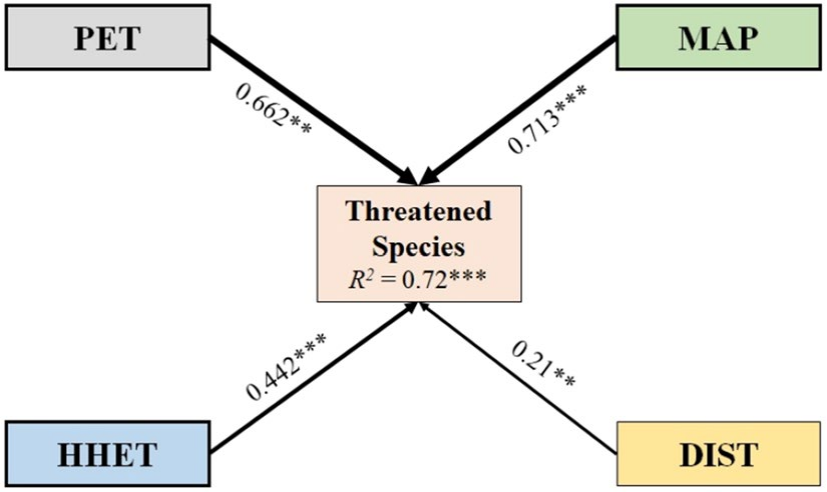
Structural equation model (SEM) showed the pathways explaining the significant effects of climate, habitat heterogeneity, and human influence on the threatened species richness. The number in the arrow and its thickness denotes the standardized partial regression coefficient. Significance: *p < 0.05; **p < 0.005; ***p < 0.001. Abbreviations are in Table 1.

SEM showed better goodness-of-fit (*R*^2^) with four impact variables, namely, PET, MAP, HHET, and DIST. All these variables showed a significant relationship in determining the richness patterns of threatened plant species in Sichuan. The most prominent variable was MAP (71.3%), followed by PET (66.2%). However, the impact of HHET and DIST was less than 50%. The overall result of SEM revealed that the impact variables significantly explained 72% of variations in threatened plant species richness in Sichuan (Fig. 4).

## Discussion

This study determined the relationships between threatened plant species richness along elevation gradients in Sichuan. The province harbored 137 species of threatened plants distributed between 200 and 4800 m above sea level. The species richness showed a hump-shaped pattern, where the highest number of species was reported at 1100 m. The species richness abruptly declined towards the end of the elevation gradient. Similar to this study, previous studies conducted in mountainous regions of the world for example—Hengduan Mountains region^34^ and Mt. Namjagbarwa region, China^35^, Mt. Kenya, Kenya^36^, Los Tuxtlas, Mexico^37^, and the Himalayas also showed a hump-shaped species richness pattern of plant species along elevation gradients^11,14,38,39^. These regions resemble similar climatic and topographic characteristics with that of Sichuan, therefore, the findings of the present study agreed with the findings of most studies in the mountainous region around the world. A high richness of threatened species was found at a low elevation band (1100 m). Two possible reasons could explain the change in threatened plant species richness. First, over-exploitation by urbanization and human pressure in the lower elevation may have resulted in highly threatened plant species at lower belt^1^. Second, a decline in species along the higher elevation could be explained by the decrease in the surface area along the elevation and change in climatic conditions^40^.

The distribution patterns of threatened plant species at spatial scales were determined. Central and Southwestern Sichuan were found to be rich in threatened species. The results also showed the Central region as a possible hotspot of threatened plant species. The distribution pattern of threatened plants in Sichuan also followed similar trends as other plant groups, such as gymnosperms and orchids, studied in China^3,5,41^.

The MDE is a common hypothesis to address the pattern of species richness of plants in mountainous areas. The MDE was introduced to address the complexity of climatic and non-climatic factors on richness patterns^21,22,42^. Some studies supported the MDE in other regions of China^23,25^. However, the MDE was found to be the least expressed variable in the richness pattern of threatened species (Fig. 1b). Paudel et al.^1^ and Wang et al.^24^ explained the least effect of the MDE in the richness pattern of threatened plant species in Nepal and Ericaceae in Yunnan Province, China, respectively. In the present study, the climatic variables were more influential than the MDE, and this phenomenon may have suppressed the MDE. Meanwhile, the highest richness of species at lower elevation instead of mid-elevation possibly demonstrated the least impact of the MDE on richness pattern.

Climatic variables have a strong influence on the richness pattern of threatened plants in Sichuan. PET was the dominant climatic variable, while MAT and MAP were equally significant for the richness pattern of threatened plant species (Table 1). PET as an individual variable has higher explanatory power for the richness pattern of threatened plants in Sichuan. It is a surrogate of net atmospheric energy balance^11,13,43^. A positively significant relationship between PET and threatened plant species denotes consistency with the productivity hypothesis, which states that greater availability of energy could enhance the species richness in area^16^. PET was used as a surrogate of net available energy estimated using temperature, water, and solar radiation^44^. In previous studies, PET explained 33.87% deviance in the richness pattern of vascular plants in Nepal^11^, 35.6% in seeded plants in the Western Himalaya^45^, 48% deviance in woody plant species in the Iberian Peninsula^46^, and 75% in gymnosperm richness in Southwestern China^38^. Therefore, the Central region of Sichuan harboring a high diversity of threatened plant species could be directly related to the high PET of the region (Fig. 3c).

In accordance with the species–temperature relationship^12^, a strong effect of MAT was found on the richness pattern of threatened plant species in Sichuan, explaining 66.22% of the variation (Table 1). Temperature is the primary source of energy available to plants^13,47^. Ambient temperature determines the availability of resources that could be effectively used by the producers (plants) in an ecosystem. In the present study, a positive relationship was found between threatened plant species and temperature. This finding is consistent with that of previous studies on threatened species in protected areas of Finland^48^ and threatened gymnosperms in China^38^. However, the temperature was the least expressed variable in the richness pattern of orchids^49^, *Rhododendron*^50^, and Gesneriaceae^29^ in China. In general, high temperatures harbor high species richness. The number of threatened species was less in the western part of the province because the region was covered by mountains and experienced low temperatures than the eastern region (Figs. 2 and 3a). These findings could be closely related to ambient energy hypothesis^51^, which states that the distribution of species is mainly determined by the physiological tolerance of species to extreme cold and hot temperatures^52^. Moreover, fewer species tend to adapt in the mountainous region due to the harsh climate condition and the decrease in optimal temperature that is required for growth and development.

The findings presented here showed that MAP was the third-best predictor that explained the highest deviance in the richness pattern of threatened plants (Table 1). Moreover, a significantly positive relationship was found between precipitation and species richness. Feng et al.^53^ also reported a positive relationship between endemic plants and precipitation in China. Similar findings were reported by Zhang et al.^54^ in all plant groups in nature reserves across Shandong Province in China. A possible explanation for the strong relationship between precipitation and species richness is that the eastern region of Sichuan receives abundant rainfall that helps threatened plant species to thrive in the study area (Fig. 3b). Moreover, the highest number of species was recorded where the annual precipitation in the region was also high (1530 mm/year). Precipitation implies the availability of water and moisture to the plant, which significantly influences the species richness of plants in general^16,20^. Moreover, the contribution of the temperature and precipitation could not be discriminated as the temperature is also considered an important predictor that determines the richness pattern at the lower-elevation region, while the precipitation profoundly explained the richness pattern at a higher elevation. Meanwhile, fluctuation in temperature and precipitation due to climate change was noticeable in the Southwestern region of China^30^. Therefore, the threatened species restricted to higher elevation were subjected to extinction due to climate change, consistent with the findings on the extinction risk assessment of threatened montane conifers^8^.

A significantly negative relationship was found between DISTs and the richness pattern of threatened plant species in Sichuan (Table 1). Various factors, such as human population density, settlements, urbanization, and land-use changes were used as a surrogate to determine the anthropogenic disturbances in the area^55^. Zhang et al.^49^ identified Southwestern China as a hotspot of plant diversity, including threatened plants. Meanwhile, Sichuan is home to large concentrations of threatened species after Yunnan, and it is particularly important for biodiversity conservation^3,49^. Similarly, Shrestha et al.^4^ reported high vulnerability of plant species in Sichuan. Their study summarized the direct influence of human pressure on the local plant population. Moreover, Panda et al.^45^ found anthropogenic disturbances as a key factor determining the species richness pattern of plants in the Western Himalayas. An increase in threatened species richness may result in habitat loss and fragmentation in human-dominated areas^56,57^. The impact of human activities and the current climate change could be more severe in species-rich areas, such as Sichuan, which could result in a high risk of species extirpation in this region^4^. Therefore, anthropogenic disturbances on threatened species should not be underestimated, indicating that the habitat loss due to anthropogenic activities may mount additional pressures on species that are on the verge of extinction.

This study presents the richness pattern of threatened plants in Sichuan by using climatic, habitat heterogeneity, and disturbances as driving factors. The study highlighted the presence of rich diversity of threatened species in Central Sichuan. The climatic factors particularly precipitation and potential evapotranspiration explain the highest deviance in the richness patterns of threatened plants. Moreover, a strong relationship between threatened plants and climatic variables shows the susceptibility of species to climate change. The comparative analysis presented here implies the increase in human influence, which may raise the alarm in the distribution and diversity of threatened plants. Therefore, the results of this study emphasize the consideration of climatic and anthropogenic factors for the in-situ conservation of threatened plant species in Sichuan. Further, future research could prioritize the protection strategies of threatened plant species in their natural habitat.

## Materials and methods

### Study area

Sichuan Province is rich in terms of ecological and biological diversity. The province is located in Southwestern China (between 26°03′N–34°19′N and 92°21′E–108°12′E), covering an area of more than 486,000 km^2^ (http://bzdt.ch.mnr.gov.cn/index.html; accessed on November 2021). The elevation of the province ranges between 188 (Wenwu Village) and 7556 (Mount Gonga) m.a.s.l. (Fig. 1a). In general, the eastern regions of Sichuan Province experience high temperatures and precipitation compared with the western region. The province is composed of mountains, hills, plains, basins, and plateaus, showing heterogeneity in habitat^26^. The vegetation in Sichuan could broadly be divided into the humid evergreen broad-leaved forest in the East Sichuan Basin region; dry evergreen broad-leaved forest in the Southwest Sichuan mountainous region; coniferous forest in the West Sichuan mountain canyon region; original coniferous forest, shrub, and meadow in the West Sichuan mountainous region; subalpine to alpine shrub and meadows in the Northwest Sichuan Plateau region^26^.

### Elevational and geographical distribution data of threatened plants

Initially, the threatened plant species reported from Sichuan were listed following Chéng^58^. This book is the most updated and reliable for cataloging the threatened plant species found in Sichuan. Further, the online portal of “Catalogue of Life” (https://www.catalogueoflife.org/; accessed between September 2021 and May 2022) was used to validate the name of species for nomenclature and synonyms. A total of 137 threatened plant species were found in Sichuan, and they were grouped into 63 taxonomic families (Supplementary Table S1). The data collected included two types, (i) Elevational distribution data were collected to determine the MDE. The maximum and minimum elevational range distribution of the aforementioned plant species were extracted from Wu and Raven^59^ and Chéng^58^. Some species lack information related to the elevational range. Therefore, the missing information was collected from the online portal of the Chinese Virtual Herbarium (http://www.cvh.ac.cn/; accessed between August 2021 and December 2021). The herbarium samples that were reported only from Sichuan were included. (ii) Geographical distribution data were also collected to determine the effect of impact variables on species richness. The geo-reference distribution of the 134 threatened species (information of about three species were not found) was obtained from Chinese Virtual Herbarium (http://www.cvh.ac.cn/; accessed between August 2021 and January 2022) and published literature. The database consisted of 1312 occurrence records in total.

### Estimation of species richness

The term species richness was used to denote the number of species present in area^6^. Here, the richness pattern of threatened plants along the elevation gradient and at a spatial resolution of 20 km × 20 km was calculated.

The interpolation method was used to estimate species richness at each elevation band to examine the relationship between species richness and elevation^60^. The elevational variation of Sichuan ranges between 188 and 7566 m.a.s.l (Fig. 1a). Initial findings suggested that the threatened species in Sichuan were distributed between 200 and 4800 m. Following Sanders^60^ and Vetaas and Grytnes^43^, the elevation range of Sichuan was differentiated from 200 and 4800 m into 47 zones of 100 m elevation bands. The interpolation method helped account for the underestimation of richness. According to this method, a species is assumed to be present in each 100-m elevational interval between its upper and lower elevational limits^11,43^. For example, *Fagus chienii* has a distribution range between 1300 and 1700 m (Supplementary Table S1). Therefore, following the interpolation method, the species was assumed to be present between 1300, 1400, 1500, 1600, and 1700 m elevation bands.

For evaluation of the richness pattern of threatened plants in the spatial area, the geographical regions of Sichuan were mapped using the same projection, and a grid cell of 20 km × 20 km of spatial resolution was overlaid using ArcGIS (version 10.3.1). Species richness was calculated as the number of species in each grid. The distribution of threatened plants was transferred into grids at a resolution of 20 km × 20 km^5,29,50^. In total, there were 1427 grids, where the species were present in 223 grid cells. Following Liu et al.^29^ and Shrestha et al.^50^, the grid cells with < 50% of spatial area cover were excluded from analyses. Moreover, to determine the species richness hotspots, the complementary algorithm was applied^29^. This algorithm was designed in a manner that it first selects grid cells with the highest species richness and then, searches for grid cells with the next highest number of species that are not found in the selected ones. The process is repeated until all species are included. The grid with the highest number of species is determined as the species richness hotspot.

### The mid-domain effect (MDE)

The MDE explains mountain geometry constraints^21,22^. RangeModel (version 5) was used to test the MDE in the present study^61,62^. It helps to generate the mean predicted species richness pattern under pure “geometric constraints”. It also uses the total number of species, the number of elevational bands, and the range size-frequency distribution data to predict the null model. A total of 10,000 Monte Carlo simulations of empirical range size sampled without replacement was run to generate the mean expected species richness and the 95% confidence interval for each elevation band. The mean expected species richness was used as an explanatory variable in the regression model to determine the effect of the MDE.

### Impact variables

Five variables, namely, three climatic, one habitat heterogeneity, and one disturbance variable were used as a possible determinants of the large-scale pattern of threatened plant species in Sichuan. The climatic variables include the mean annual temperature (MAT), mean annual precipitation (MAP), and PET^6,8,11,29,38,45,51^. HHET was used as a range of elevation^18^. The Human Footprint Index (HFI) was utilized as the surrogate of the level of disturbance (DIST)^63,55^. All the rasters of impact variables are available at different spatial resolutions. Therefore, by following, Liu et al.^29^ and Shrestha et al.^50^, all the variables were transformed into a resolution of 20 km × 20 km in ArcGIS (version 10.3.1) by calculating the average of all data points within each grid cell. The “resample” function in ArcGIS helped in performing the calculations.

### Climate

Previous studies have shown that species richness is highly influenced by energy and water^5,12,19^. Temperature and precipitation are considered the surrogate of energy and humidity-related climatic factors. Therefore, in the present study, MAT (°C/year) and MAP (mm/year) were downloaded from CHELSA 1.2, available at 1 km resolution (at the equator^64,31^; http://chelsa-climate.org/bioclim/) (Fig. 3a,b). PET (mm/year) estimates the net atmospheric energy balance with respect to water availability^11,13^. Temperature, precipitation, and solar radiation were used to calculate PET in specific areas. PET was downloaded from MODIS Global Evapotranspiration Project (MOD16) available at 0.5 km resolution at the equator^65,32^ (http://www.ntsg.umt.edu/project/modis/mod16.php) as shown in Fig. 3c. The evapotranspiration algorithm used in MOD16 was based on the Penman–Monteith equation.

### Habitat heterogeneity

HHET (m) was measured by the range of elevation. Previous studies have shown a significant effect of range of elevation in the richness pattern of *Rhododendron*^50^ and orchid^66^ in China, and woody plant richness in Iberian Peninsula^46,50^. Due to similarities in the tropography of the aforementioned study sites with Sichuan, the range of elevation is used as a surrogate of HHET. It was used to represent topographic relief which was calculated as the difference between the maximum and minimum elevation of a grid cell^50^. HHET The digital elevation model was downloaded from the NASA-Jet Propulsion Laboratory portal^28^ (https://lpdaac.usgs.gov/products/srtmgl1v003/) and available at 1 arc sec (30 m) resolution at the equator (Fig. 1a).

### Disturbances

Studies have revealed that human-induced effects are a major threat to biodiversity^1,56,55^. Human footprint refers to the direct and indirect pressures from human activities on the environment. Therefore, as a surrogate of anthropogenic disturbance, a quantitative measurement index, such as HFI was frequently used in previous studies^63,55^. Mu et al.^55^ mapped the annual dynamics of the global HFI from 2000 to 2018 by using eight human pressure variables (i.e., population density, navigable waterways, roads, railways, pasture, nighttime light, built environment, and cropland). In the present study, two time series (first and last) were used to calculate the impact of disturbances on the environment, with the expectation that the changes in habitat are noticeable in these two time series. DIST variable was calculated as the difference in annual HFI between 2018 and 2000 (Fig. 3d). These changes could be positive and/or negative, with positive representing undisturbance or restored habitat and negative representing the disturbance over a period of time. The data were available at 1 km resolution (at the equator) and downloaded from the source provided in Mu et al.^55,33^. The “raster calculator” function in ArcGIS was used to calculate the degree of DIST at the required spatial scale.

### Statistical analyses

A two-step procedure was used to determine the relationship between species richness and the impact variables. First, the direct relationship between species richness and elevation was analyzed. Second, the relationship between species richness and impact variables was evaluated. Polynomial regression was performed to define *richness–elevation* relationship. The *R*^2^ value of the regression was used to determine the goodness-of-fit of a model^67^.

The species richness data were overdispersed, i.e., the variance exceeded the mean. Therefore, to examine the direct effect of impact variables (MAT, MAP, PET, HHET, and DIST) on species richness, the generalized linear model (GLM) with negative binomial regression distribution was used. Negative binomial regression is widely used for overdispersed count data^68^. Stepwise GLM regression is used to select the best variable combination that determines the richness pattern of threatened plant species^69^. Both regressions were conducted in the present study to identify the best model with all the possible combinations of five impact variables. In total, 31 models were generated, with possible combination of 1–5 impact variables each. Akaike information criterion (AIC) and adjusted (*Adj*) *R*^2^ were used to assess the goodness-of-fit of the regression model to select which impact variables should be included in the final model^67^. The best predictor was selected based on high *Adj R*^2^ and low AIC value. Variance inflation factor (VIF) was used to check the multicollinearity among the impact variables in the model. Such multicollinearity was considered significant when VIF > 5. Therefore, the best model included the variable with VIF < 5.

Structural equation modeling (SEM) was also implemented to evaluate the potential causal relationship between species richness and predictor variables. SEM uses regression and path analyses for the significance of the overall model structure that is suitable to evaluate the species–predictor relationship^70,71^. As all predictor variables potentially mediate the species richness pattern, the effects of individual variables and all the variables together on the species richness of threatened plants were tested^70,71^. Given that the MDE in the mountain domains is independent of any environmental gradients^21,22^, the MDE was excluded in the regression and path analysis.

**Figure 5:**
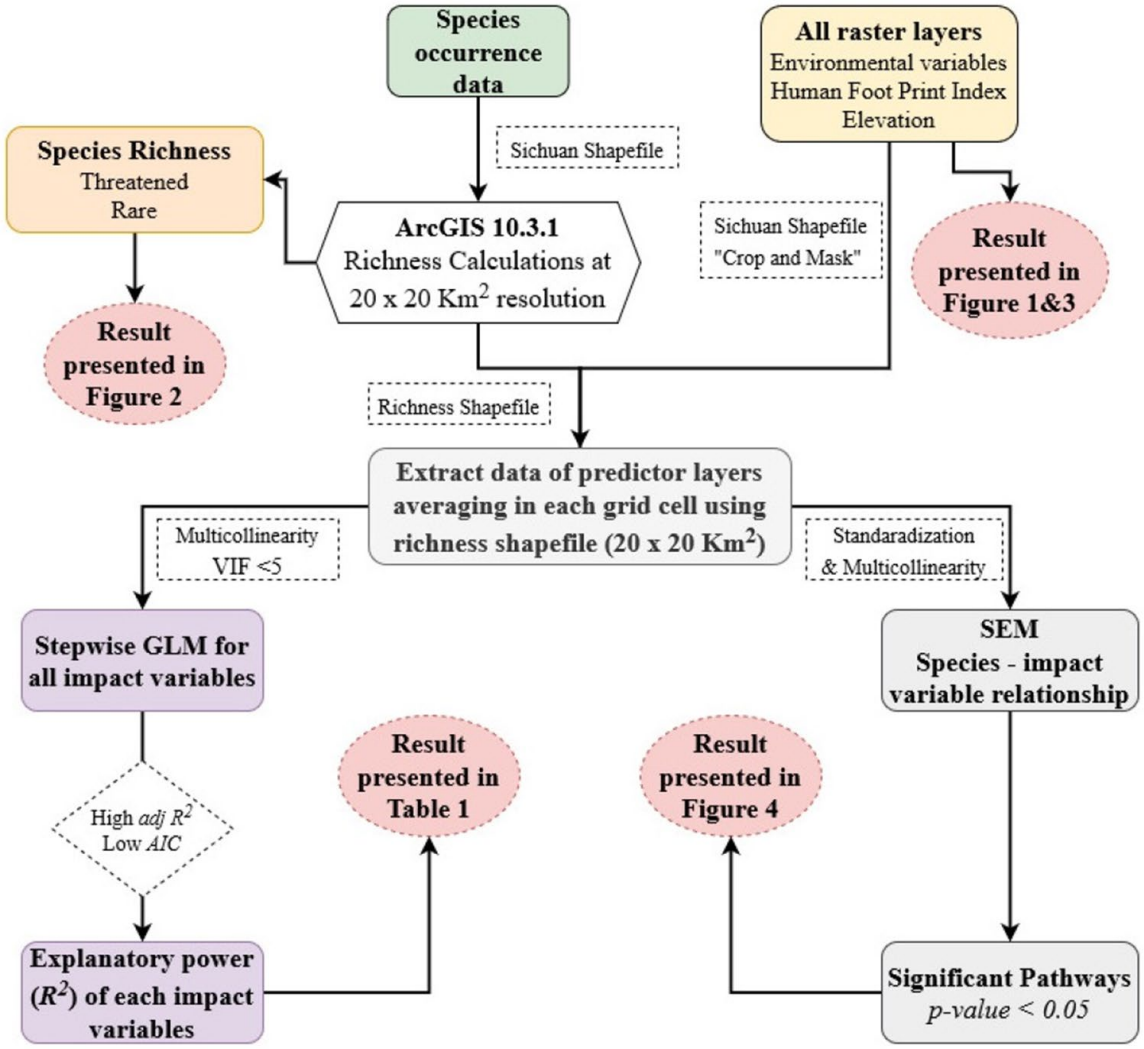
Flowchart showing methodological framework.

All the analyses were performed in R version 4.0.1^72^. “MASS” package was used for GLM regression^73^, “car” package was used to check the VIF of variables in each model^74^, and “lavaan” package was used for SEM^75^. The flowchart showing the methodological framework is represented in Fig. 5

.

## Supplementary Information

Supplementary Table S1.

## Data Availability

All datasets for this study were downloaded from the open sources archives (mentioned in the text). The list of species and their elevation distribution are included in the supplementary material. Additionally, the geographical distribution points of species used during and/or analysed during the current study are available from the corresponding author on reasonable request.
