# Supplementary material for: Precipitation and potential evapotranspiration determine the distribution patterns of threatened plant species in Sichuan Province, China
Source: Sci Rep. 2022 Dec 27;12:22418. doi: 10.1038/s41598-022-26171-5 (PMC9794706; doi:10.1038/s41598-022-26171-5)
Supplement: Supplementary file 1 — Supplementary Table S1. [file 41598_2022_26171_MOESM1_ESM.docx]

Supplementary material

**Supplementary Table S1:** List of species with respective families and elevation range. The elevation range represents the lower and higher elevation bands of respective species expressed in meter (above sea level).

| **Name of Species** | **Family** | **Elevation range (m)** | |
| --- | --- | --- | --- |
|  |  | **Low** | **High** |
| *Acanthochlamys bracteata* P.C.Kao | Acanthochlamydaceae | 2700 | 3500 |
| *Acer japonicum* C. P. Thunberg ex A. Murray | Aceraceae | 400 | 1000 |
| *Dipteronia sinensis* Oliver | Aceraceae | 1000 | 2000 |
| *Acer pentaphyllum* Diels | Aceraceae | 2300 | 2900 |
| *Nouelia insignis* Franch. | Asteraceae | 1000 | 2500 |
| *Podophyllum versipelle* subsp. Versipelle | Berberidaceae | 300 | 2400 |
| *Podophyllum hexandrum* Royle | Berberidaceae | 2200 | 4300 |
| *Corylus chinensis* Franch. | Betulaceae | 2000 | 3500 |
| *Bretschneidera sinensis* Hemsl. | Bretschneideraceae | 300 | 1700 |
| *Brasenia schreberi* J. F. Gmel. | Cabombaceae | 400 | 1500 |
| *Psammosilene tunicoides* W. C. Wu & C. Y. Wu | Caryophyllaceae | 2000 | 3800 |
| *Cephalotaxus oliveri* Mast. | Cephalotaxaceae | 300 | 1800 |
| *Cibotium barometz* (L.) J. Sm. | Cibotiaceae | 100 | 1600 |
| *Cercidiphyllum japonicum* Siebold & Zucc. | Crecidiphyllaceae | 650 | 2700 |
| *Fokienia hodginsii* (Dunn) A. Henry & H. H. Thomas | Cupressaceae | 100 | 1800 |
| *Cupressus chengiana* var. *jiangensis* (N. Zhao) Silba | Cupressaceae | 1000 | 2500 |
| *Cupressus chengiana* S. Y. Hu | Cupressaceae | 1200 | 2900 |
| *Thuja sutchuenensis* Franch. | Cupressaceae | 1400 | 4710 |
| *Alsophila spinulosa* (Wall. ex Hook.) R. M. Tryon | Cyatheaceae | 260 | 1600 |
| *Alsophila acaulis* Mak. | Cyatheaceae | 300 | 1500 |
| *Alsophila metteniana* Hance | Cyatheaceae | 300 | 1500 |
| *Cycas szechuanensis* W.C.Cheng & L.K.Fu | Cycadaceae | 400 | 1300 |
| *Cycas panzhihuaensis* L.Zhou & S.Y.Yang | Cycadaceae | 1100 | 2000 |
| *Cystopteris chinensis* (Ching) Wei & Zhang | Cystopteridaceae | 200 | 2500 |
| *Polystichum glaciale* Christ | Dryopteridaceae | 3200 | 4700 |
| *Rhododendron rex* H. Lév. | Ericaceae | 2300 | 3300 |
| *Rhododendron rex* subsp. *fictolacteum* (I. B. Balf.) D. F. Chamb. | Ericaceae | 2900 | 4000 |
| *Rhododendron cyanocarpum* (Franch.) W. W. Sm. | Ericaceae | 3000 | 4000 |
| *Eucommia ulmoides* Oliv. | Eucommiaceae | 300 | 500 |
| *Euptelea pleiosperma* Hook. fil. & Thomson | Eupteleaceae | 900 | 3600 |
| *Ormosia henryi* Prain | Fabaceae | 100 | 1300 |
| *Glycine max* subsp. *soja* (Siebold & Zucc.)H.Ohashi | Fabaceae | 150 | 2650 |
| *Ormosia hosiei* Hemsl. & E.H.Wilson | Fabaceae | 200 | 900 |
| *Ormosia nuda* (F.C.How)R.H.Chang & Q.W.Yao | Fabaceae | 800 | 2000 |
| *Eucheresta japonica* Hook. f. ex Regel. | Fabaceae | 800 | 1350 |
| *Salweenia bouffordiana* H.Sun, Zhi M.Li & J.P.Yue | Fabaceae | 2700 | 3600 |
| *Fagus hayatae* Palib. ex Hayata | Fagaceae | 1300 | 2300 |
| *Fagus chienii* W.C.Cheng | Fagaceae | 1300 | 1700 |
| *Myriophyllum ussuriense* (Regel) Maxim. | Haloragaceae | 300 | 1800 |
| *Liquidambar chingii* (F. P. Metcalf) Ickert-Bond & J. Wen | Hamamelidaceae | 1000 | 1100 |
| *Sinowilsonia henryi* Hemsl. | Hamamelidaceae | 1100 | 1600 |
| *Ottelia acuminata* (Gagnep.) Dandy | Hydrocharitaceae | 800 | 1965 |
| *Ottelia acuminata* var. *crispa* (Hand.-Mazz.) H.Li | Hydrocharitaceae | 800 | 1500 |
| *Isoetes hypsophila* Hand.-Mazz. | Isoetaceae | 3500 | 4300 |
| *Juglans mandshurica* Maxim. | Juglandaceae | 350 | 1550 |
| *Juglans regia* L. | Juglandaceae | 400 | 1800 |
| *Cinnamomum camphora* (L.) J. Presl | Lauraceae | 510 | 1100 |
| *Phoebe zhennan* S. K. Lee & F. N. Wei | Lauraceae | 550 | 1900 |
| *Cinnamomum longepaniculatum* (Gamble) N. Chao ex H. W. Li | Lauraceae | 600 | 2000 |
| *Machilus nanmu* (Oliv.) Hemsl. | Lauraceae | 820 | 1000 |
| *Cinnamomum mairei* H. Lév. | Lauraceae | 1300 | 1800 |
| *Trillium tschonoskii* Maxim. | Liliaceae | 1600 | 3200 |
| *Heteropolygonatum pendulum* (Z.G.Liu & X.H.Hu) M.N.Tamura & Ogisu | Liliaceae | 2000 | 2200 |
| *Magnolia officinalis* Rehder & E.H.Wilson | Magnoliaceae | 300 | 1500 |
| *Magnolia ernestii* Figlar | Magnoliaceae | 600 | 2000 |
| *Magnolia patungensis* (Hu) Noot. | Magnoliaceae | 600 | 1000 |
| *Magnolia insignis* Wall. | Magnoliaceae | 900 | 1200 |
| *Liriodendron chinense* (Hemsl.) Sarg. | Magnoliaceae | 900 | 1000 |
| *Magnolia omeiensis* (W.C.Cheng) Dandy | Magnoliaceae | 1200 | 1300 |
| *Magnolia dawsoniana* Rehder & E.H.Wilson | Magnoliaceae | 1400 | 2500 |
| *Magnolia sieboldii* subsp. *sinensis* (Rehder & E.H.Wilson) Spongberg | Magnoliaceae | 1680 | 2600 |
| *Magnolia wilsonii* (Finet & Gagnep.) Rehder | Magnoliaceae | 1900 | 3300 |
| *Toona ciliata* M. Roem. | Meliaceae | 800 | 1500 |
| *Toona ciliata* var. *pubescens* (Franch.) Hand.-Mazz. | Meliaceae | 850 | 1200 |
| *Camptotheca acuminata* Decne. | Nyssaceae | 460 | 1000 |
| *Davidia involucrata* Baill. | Nyssaceae | 1500 | 2200 |
| *Syringa pinnatifolia* Hemsl. | Oleaceae | 2600 | 3100 |
| *Ophioglossum thermale* Komarov | Ophioglossaceae | 100 | 3000 |
| *Gastrodia elata* Blume | Orchidaceae | 400 | 3200 |
| *Changnienia amoena* S.S.Chien | Orchidaceae | 400 | 1800 |
| *Holcoglossum omeiense* X.H.Jin & S.C.Chen | Orchidaceae | 700 | 1000 |
| *Cypripedium micranthum* Franch. | Orchidaceae | 2000 | 2500 |
| *Cypripedium palangshanense* Tang & F.T.Wang | Orchidaceae | 2200 | 2700 |
| *Cypripedium margaritaceum* Franch. | Orchidaceae | 2500 | 3600 |
| *Cypripedium lichiangense* S.C.Chen & P.J.Cribb | Orchidaceae | 2600 | 3500 |
| *Paeonia delavayi* Franch. | Paeoniaceae | 2300 | 3700 |
| *Paeonia delavayi* subsp. *lutea* (Delavay ex Franch.) B. A. Shen | Paeoniaceae | 2300 | 3700 |
| *Paeonia suffruticosa* Andrews | Paeoniaceae | 2400 | 3100 |
| *Meconopsis punicea* Maxim. | Papaveraceae | 2800 | 4300 |
| *Ceratopteris thalictroides* (L.) Brongn. | Parkeriaceae | 300 | 1000 |
| *Pseudotsuga sinensis* Dode | Pinaceae | 800 | 2800 |
| *Picea neoveitchii* Mast. | Pinaceae | 1300 | 2000 |
| *Picea brachytyla* (Franch.) E. Pritz. | Pinaceae | 1500 | 3800 |
| *Pseudotsuga sinensis* var. *sinensis* | Pinaceae | 1700 | 3300 |
| *Picea brachytyla* var. *complanata* (Mast.) W. C. Cheng ex Rehd. | Pinaceae | 2000 | 3800 |
| *Tsuga forrestii* Downie | Pinaceae | 2000 | 3000 |
| *Larix mastersiana* Rehd. & E.H. Wilson | Pinaceae | 2300 | 3500 |
| *Abies chensiensis* Tiegh. | Pinaceae | 2300 | 3000 |
| Picea brachytyla var. aurantiaca | Pinaceae | 2600 | 3600 |
| Picea brachytyla var. montigena | Pinaceae | 2600 | 3300 |
| *Abies forrestii* var. *georgei* (Orr) Farjon | Pinaceae | 3400 | 4200 |
| *Sorghum propinquum* (Kunth) Hitchc. | Poaceae | 350 | 1000 |
| *Chimonobambusa tumidissinoda* Ohrnb. | Poaceae | 1500 | 2200 |
| *Elymus hitchcockii* Davidse | Poaceae | 2000 | 3500 |
| *Cenchrus sichuanensis* (S.L.Chen & Y.X.Jin) Morrone | Poaceae | 2000 | 3000 |
| *Elymus sinosubmuticus* S.L.Chen | Poaceae | 2500 | 3000 |
| *Fagopyrum acutatum* (Lehm.) Mansf. ex K. Hammer | Polygonaceae | 250 | 3200 |
| *Neocheiropteris palmatopedata* (Bak.) Christ | Polypodiaceae | 1500 | 2700 |
| *Pomatosace filicula* Maxim. | Primulaceae | 3000 | 4500 |
| *Aleuritopteris grevilleoides* (Christ) G. M. Zhang & X. C. Zhang | Pteridaceae | 1100 | 1800 |
| *Coptis chinensis* Franch. | Ranunculaceae | 500 | 2000 |
| *Urophysa rockii* Ulbr. | Ranunculaceae | 600 | 800 |
| *Coptis omeiensis* (Chen) C. Y. Cheng | Ranunculaceae | 1000 | 1700 |
| *Kingdonia uniflora* Balf. fil. & W. W. Sm. | Ranunculaceae | 2750 | 3900 |
| *Aconitum brachypodum* Diels | Ranunculaceae | 2800 | 3700 |
| *Circaeaster agrestis* Maxim. | Ranunculaceae | 3200 | 4150 |
| *Rhoiptelea chiliantha* Diels & Handel-Mazzetti | Rhoipteleaceae | 700 | 2500 |
| *Potentilla omeiensis* (T. T. Yu & C. L. Li) Soják | Rosaceae | 2000 | 4800 |
| *Malus sikkimensis* (Wenz.) Koehne | Rosaceae | 2500 | 3000 |
| *Emmenopterys henryi* Oliv. | Rubiaceae | 400 | 1600 |
| *Phellodendron chinense* C. K. Schneid. | Rutaceae | 900 | 2500 |
| *Salix magnifica* Hemsl. | Salicaceae | 2100 | 2800 |
| *Litchi chinensis* Sonner. | Sapindaceae | 300 | 800 |
| *Scheuchzeria palustris* L. | Scheuchzeriaceae | 1700 | 2080 |
| *Picrorhiza scrophulariiflora* Pennell | Scrophulariaceae | 3600 | 4400 |
| *Anisodus tanguticus* (Maxim.) Pascher | Solanaceae | 2800 | 4200 |
| *Paradombeya sinensis* Dunn | Sterculiaceae | 280 | 1500 |
| *Firmiana major* (W. W. Sm.) Hand.-Mazz. | Sterculiaceae | 1600 | 3000 |
| *Pterostyrax psilophyllus* Diels ex Perkins | Styracaceae | 600 | 2500 |
| *Rehderodendron macrocarpum* Hu | Styracaceae | 1000 | 1500 |
| *Myricaria laxiflora* (Franch.) P. Y. Zhang & Y. J. Zhang | Tamaricaceae | 300 | 2800 |
| *Tapiscia sinensis* Oliver | Tapisciaceae | 400 | 1800 |
| *Amentotaxus argotaenia* (Hance) Pilg. | Taxaceae | 300 | 1100 |
| *Torreya fargesii* Franch. | Taxaceae | 1000 | 3400 |
| *Taxus mairei* (Lemée & Lév.) S. Y. Hu ex T. S. Liu | Taxaceae | 1350 | 3500 |
| *Taxus yunnanensis* W. C. Cheng & L.K. Fu | Taxaceae | 2000 | 3500 |
| *Torreya grandis* var. *grandis* | Taxaceae | 2100 | 2300 |
| *Taxus wallichiana* var. *chinensis* (Pilg.) Florin | Taxaceae | 2300 | 3100 |
| *Taxus wallichiana* Zucc. | Taxaceae | 2500 | 3000 |
| *Glyptostrobus pensilis* (Staunton ex D. Don) K. Koch | Taxodiaceae | 550 | 1000 |
| *Cunninghamia lanceolata* (Lamb.) Hook. | Taxodiaceae | 1500 | 2100 |
| *Tetracentron sinense* Oliver | Tetracentraceae | 1500 | 2500 |
| *Stewartia sinensis* Rehder & E. H. Wilson | Theaceae | 600 | 1900 |
| *Camellia luteoflora* Y. K. Li ex H. T. Chang & F. A. Zeng | Theaceae | 600 | 1500 |
| *Trapa incisa* Sieb. & Zucc. | Trapaceae | 300 | 1500 |
| Pteroceltis tatarinowii *Maxim.* | Ulmaceae | 100 | 1500 |
| *Zelkova schneideriana* Hand.-Mazz. | Ulmaceae | 200 | 1100 |
